# Emerging Roles of Impaired Autophagy in Fatty Liver Disease and Hepatocellular Carcinoma

**DOI:** 10.1155/2021/6675762

**Published:** 2021-04-22

**Authors:** Suryakant Niture, Minghui Lin, Leslimar Rios-Colon, Qi Qi, John T. Moore, Deepak Kumar

**Affiliations:** ^1^Julius L. Chambers Biomedical Biotechnology Research Institute, North Carolina Central University Durham, NC 27707, USA; ^2^The Fourth People's Hospital of Ningxia Hui Autonomous Region, Yinchuan, China 750021

## Abstract

Autophagy is a conserved catabolic process that eliminates dysfunctional cytosolic biomolecules through vacuole-mediated sequestration and lysosomal degradation. Although the molecular mechanisms that regulate autophagy are not fully understood, recent work indicates that dysfunctional/impaired autophagic functions are associated with the development and progression of nonalcoholic fatty liver disease (NAFLD), alcoholic fatty liver disease (AFLD), and hepatocellular carcinoma (HCC). Autophagy prevents NAFLD and AFLD progression through enhanced lipid catabolism and decreasing hepatic steatosis, which is characterized by the accumulation of triglycerides and increased inflammation. However, as both diseases progress, autophagy can become impaired leading to exacerbation of both pathological conditions and progression into HCC. Due to the significance of impaired autophagy in these diseases, there is increased interest in studying pathways and targets involved in maintaining efficient autophagic functions as potential therapeutic targets. In this review, we summarize how impaired autophagy affects liver function and contributes to NAFLD, AFLD, and HCC progression. We will also explore how recent discoveries could provide novel therapeutic opportunities to effectively treat these diseases.

## 1. Introduction

**Figure 1:**
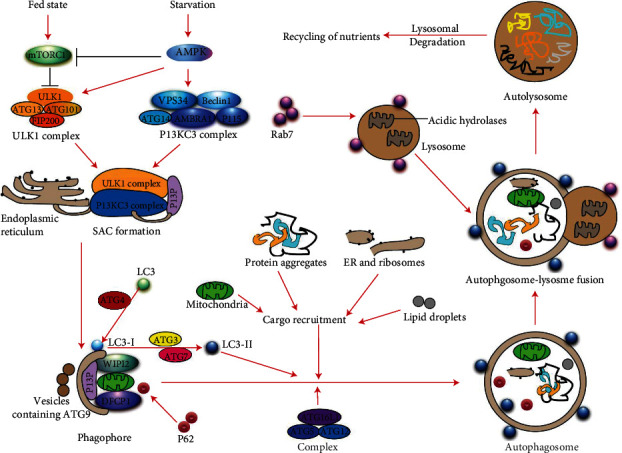
Schematic model represents the overview of the autophagy process. Molecular regulation of the autophagic process in normal and starvation conditions is presented. In the presence of sufficient nutrients, activation of mTOR inhibits the ULK1 complex, whereas, under conditions of nutrient starvation, AMPK inhibits mTOR and activates the ULK1 and PI3KC3 complex leading to initiation of phagophore biosynthesis. During autophagosome maturation, cargo recruitment takes place through the recruitment of ATGs and LC3, and mature autophagosome formation occurs. Ultimately, the autophagosome fuses with a lysosome, facilitated by Rab7 and LAMP proteins. The fully functional autolysosome then degrades the autolysosomal cargo and releases the degradation products into the cytosol for recycling.

Autophagy is a cellular catabolic process that eliminates damaged cell organelles, unfolded proteins, and various intracellular pathogens through lysosomal degradation. Autophagy also regulates cell proliferation, differentiation, survival, and apoptosis, highlighting its role in maintaining cellular homeostasis [1]. In general, autophagy degrades long-lived damaged intracellular proteins, in contrast to the ubiquitin-proteasome system, which controls the degradation of short-lived proteins [2]. Canonical autophagy initiates with the formation of a small vesicular sac called a phagophore (Figure 1). The phagophore encloses small portions of the cytoplasm to form a double-membrane structure called an autophagosome. Although it is still not clear where autophagosome membranes originate, possible sources are the endoplasmic reticulum (ER), mitochondria, and the Golgi [3]. Several physiological stresses including nutrient starvation, hypoxia, reactive oxygen species (ROS) production, pathogenic infections, and chemotherapy can trigger phagophore formation. During elongation and maturation, the phagophore encapsulates damaged proteins and cell organelles. Once the autophagosome forms, it fuses with the lysosome and creates a structure called the autolysosome. Lysosomal enzymes then degrade the cargo contained within the autolysosome. After the degradation of damaged proteins and lipids, amino acids and fatty acids are released into the cytoplasm and recycled for new biosynthesis of cellular components or energy production [4].

Induction of canonical autophagy is mainly controlled by the AKT/mTOR and AMPK signaling pathways and relies on the activation and assembly of two macromolecular complexes, the ATG1/ULK1 and the Class III phosphatidylinositol 3-kinase (PI3K) complex (Figure 1) [5]. First, phagophore formation requires cytoplasmic vesicle nucleation, which occurs when the ATG1/ULK1 complex, which is comprised of ULK1, FIP200, ATG13, and ATG101, interacts with the Class III PI3K complex. BECN1, ATG14, AMBRA1, VPS34, VPS15, and UVRAG proteins constitute the Class III PI3K complex. The autophagophore membrane then elongates and encloses the molecules to be degraded forming an autophagosome, which occurs in two separate conjugation reactions catalyzed by autophagy-related proteins (ATGs). In the first reaction, conjugation occurs between ATG5 and ATG7, as well as ATG10 and ATG12, which then bind to ATG16L. In the second reaction, ATG7 and ATG3, together with the ATG12-ATG5 : ATG16L complex and microtubule-associated proteins I/II light chain 3B (hereafter referred to as “LC3-I or LC3-II”), conjugate with lipid phosphatidylethanolamine (PE) to generate LC3-II. LC3 consists of a soluble form LC3-I (molecular weight 18 kDa) and a lipidated form LC3-II (molecular weight 16 kDa). The lipidation of LC-II facilitates its anchoring at the autophagosomal membrane where mature autophagosome formation occurs (Figure 1). The LC3-binding protein p62 (sequestosome-1: a multifunctional adaptor protein) binds and recruits ubiquitinated substrates to be enclosed in autophagosomes for proteasomal degradation [6]. Finally, the autophagosome fuses with a lysosome through the help of lysosomal proteins such as LAMPs and RAB7 to form the autolysosome. In the autolysosome, lysosomal acidic hydrolases, lipases, and nucleases degrade damaged proteins, lipids, and nucleic acids. The resulting degradation products such as amino acids, small fatty acids, and nucleotides are then released into the cytoplasm (Figure 1).

Researchers routinely study canonical autophagic flux through the expression of the ATG proteins, Baclin-1, and LC3-II [7]. Autophagic flux represents the entire dynamic process described above including autophagosome formation, a fusion of the autophagosome with lysosomes, and breakdown and release of autolysosome cargo components back into the cytosol [8]. Whereas, in the noncanonical autophagy process, unconventional autophagosome biogenesis occurs in the absence of some of these key autophagy proteins, using only a subset of the core ATG machinery [9].

In mammalian cells, three types of autophagy have been described: microautophagy, chaperone*-*mediated autophagy (CMA), and macroautophagy (hereafter referred to as “autophagy”). These are regarded as nonspecific degradation systems, in which a cargo of unfolded proteins, lipids, and pathogenic organisms are degraded through the autolysosome system. However, recent studies show that autophagy can also degrade specific targets, a process called “selective autophagy” [10]. This process is named after its specific targets such as mitophagy (mitochondrial cargo degradation) [11], aggrephagy (aggregated protein degradation) [12], lipophagy (lipid degradation) [13], xenophagy (pathogenic organism degradation) [14, 15], and ciliophagy (cilia component degradation) [16]. In general, activation of selective autophagy is dependent on specific vesicle trafficking pathways, secretory pathways, and additional intracellular transport processes [17].

Due to the important role of autophagy in the regulation of fundamental metabolic functions, disruption of this process can contribute to the development of a variety of diseases, including liver disease [18, 19]. NAFLD and AFLD are two of the most predominant liver diseases in the United States [20], and if unaddressed, these conditions could potentially develop into HCC [21]. Autophagy eliminates damaged cell organelles and proteins, protecting hepatocytes against toxicity/injury [22]. Autophagy not only regulates liver detoxification but also facilitates lipid degradation and thus prevents liver steatosis (fatty liver disease), an early event of both NAFLD and AFLD [22]. When lipid degradation is inhibited, particularly in chronic early NAFLD and AFLD, defective autophagy predominantly exists. As a result, impaired or defective autophagy can contribute to the development and progression of NAFLD, AFLD, and subsequent HCC. Therefore, proper modulation of autophagy can control the occurrence and outcome of liver-related diseases. In the next sections, we will discuss the role of impaired/defective autophagy in the development and progression of the NAFLD, AFLD, and HCC.

## 2. Role of Autophagy in NAFLD

Autophagy plays a central role in NAFLD pathophysiology [23]. NAFLD development begins with hepatic steatosis, characterized by increased triglyceride accumulation within hepatocytes [24]. It is estimated that 15 to 30% of liver steatosis cases progress to nonalcoholic steatohepatitis (NASH), a highly inflammatory state of the liver. NASH can subsequently progress to cirrhosis, and about 7% of cirrhotic livers develop HCC [24]. The induction of autophagic flux was reported during obesity-related NAFLD development [25]. Lipid droplets accumulated in liver cells, acting as substrates for autophagy to be degraded by autolysosomal enzymes [26]. Initially, Ohsaki et al. suggested that apolipoprotein B (ApoB) colocalized at the surface of lipid droplets in hepatocytes and is degraded by autophagy [27]. Other reports indicate that bortezomib-induced Mallory–Denk bodies in liver cells can be degraded by rapamycin-induced autophagy [28], suggesting that autophagy degrades lipids and Mallory–Denk bodies in the liver and prevents NAFLD progression. Autophagy also regulates lipid storage in hepatocytes since inhibition of autophagy by 3-Methyladenine or knockdown of *Atg5*, increased triglyceride levels in these cells [29]. During nutrient deprivation, inhibition of autophagy through the regulation of autophagic components such as ATG5 and ATG7 resulted in increased triglyceride accumulation in cultured hepatocytes and mouse liver [29]. Hepatocyte-specific knockout *ATG7* gene reduced ATG7 conjugation with ATG5 and LC3-II in the liver, leading to increased total cholesterol content in hepatocytes. The percentage of cholesterol in lysosomes was also significantly decreased upon *ATG7* knockdown, suggesting that optimal regulation of autophagy is required for lipid metabolism [29].

Autophagy also regulates hepatic inflammation. Macrophages isolated from *ATG5* knockout mice fed with a high-fat diet (HFD) and treated with low-dose lipopolysaccharide (LPS) developed systemic hepatic inflammation through regulation of macrophage polarization and due to inhibition of hepatic autophagy [30], indicating that functional autophagy modulates liver-specific macrophage polarization and reduces inflammation [30]. Moreover, impaired autophagic functions in the liver (caused by multiple conditions) contribute to a predisposition to NASH (highly inflammatory liver stage). The prevalence of NASH increases with age in both mice and humans and could be the result of decreased autophagic flux [31]. Recently, a study utilizing NASH patient samples demonstrated that more than half of liver sinusoidal endothelial cells (LSECs) show autophagic vacuoles without liver steatosis or histological abnormalities [32]. LSECs isolated from endothelial autophagy-deficient mice or mice fed with a high-fat diet showed increased expression of inflammatory pathway proteins (Ccl2, Ccl5, Il6, and VCAM-1), increased expression of endothelial-to-mesenchymal transition (EMT) markers (*α*-Sma, Tgfb1, Col1a2), and increased apoptosis, as well as perisinusoidal fibrosis in livers from endothelial autophagy-deficient animals exposed to carbon tetrachloride [32]. The study concluded that an autophagic defect occurs in NASH patients, and deficiency in endothelial autophagy promotes liver fibrosis through increased liver inflammation, EMT, and apoptosis. Mice fed with a high-fat diet (HFD) also showed suppressed autophagic flux resulting in impaired liver and kidney function [33]. Induction of hepatic steatosis inhibited autophagy in Kupffer cells leading to an increased inflammatory response when cells exposed to endotoxin [34]. Induction of hepatic steatosis also promoted disease progression by deregulating autophagic function [35] and impaired autophagy promotes steatosis in postmortem human livers [36]. Double immunofluorescence staining of adipose differentiation-related protein (ADRP) and LC3 demonstrated an inverse relationship between ADRP positive areas and LC3 positive areas, as well as the colocalization of ADRP and LC3 on a part of small lipid droplets, suggesting that impaired autophagy increased hepatic steatosis and regulates lipid droplet turnover [36]. Another study demonstrated that autophagic flux is impaired in both livers of NAFLD and NASH patients, and mouse models of NAFLD fed with a high-fat diet. They observed that overload of fatty acids resulted in a significant increase in ER stress, blockade of the autophagic flux, and apoptosis. Treatment with rapamycin, an mTOR inhibitor that activates autophagy, reduced cell death and ER stress in Huh7 cells, demonstrating the contribution of this catabolic process in cell homeostasis [37]. Increased lipid loading in lysosomes and increased p62/SQSTM1 activity also correlated with altered or impaired autophagy resulting in increased NAFLD activity. This was observed in NAFLD patients compared to healthy controls, and in both cellular and mouse models fed with a high-fat diet [38].

It is important to note the dichotomy in reports regarding the role of autophagy in NAFLD development. Several studies have concluded that autophagy has both lipolytic and lipogenic functions depending on the experimental context [23]. Lipophagy is essential for the processing of lipid droplets and the prevention of liver damage, particularly in the early stages of steatosis [39]. Inhibition of autophagy by pharmacological drugs or deregulation/knockdown of autophagic core components (ATG5, ATG7), increased hepatocyte triglyceride (TG) accumulation due to impaired lipolysis both *in vitro* and *in vivo* [29]. Reduction of ATG7 protein levels was also observed in obese mice liver samples [40]. Stimulation of autophagy by enforced expression of hepatic ATG7 positively modulated lipid metabolism and inhibited hepatic steatosis [40]. On the other hand, studies also show that autophagy can also regulate lipogenic mechanisms mostly under nutrient deficient or fasting conditions. Fasting-induced steatosis was observed in C57Bl/6 mouse liver samples and increased fat accumulation was detected in human livers after fasting for a period of 36 hours [41, 42]. Interestingly, mice with autophagy deficient-livers did not show fasting-induced steatosis. However, smaller lipid droplets and lower amount of triglyceride (TG) content was observed in these samples compared to wild type under the same fasting conditions [42]. These results suggested that autophagy enhanced lipogenesis is under fasting conditions [43]. Furthermore, hepatic knockout of *Atg7* or *Atg5* genes upregulated the production of ketone bodies in fasting mice [44]. Hepatic knockout of *Atg7* or *Atg5* genes also reduced the expression of enzymes involved in lipid oxidation. Mechanistically, the nuclear receptor corepressor 1 (NCoR1) which interacts with PPAR*α*, inhibiting its transactivation and results in increased lipid oxidation. Interaction of NCoR1 with autophagosomal GABA, RAP family proteins result in its degradation through autophagy. Loss of autophagy increased accumulation of NCoR1, reducing PPAR*α* activity, and subsequently inhibited lipid oxidation [44]. This study was concluded that functional autophagy during fasting results is in NCoR1 degradation, PPAR*α* activation, and regulation of *β*-oxidation and production of ketone bodies [44].

In summary, these studies show that autophagy can modulate both hepatic lipid degradation and lipid accumulation under different physiological conditions. Significantly, the evidence suggests that under conditions of nutrient deficiency, autophagy aids in the accumulation of lipids. It was not specified, however, if this lipid accumulation was transient, or what the long-term effects this could have in NAFLD development. Many questions remain regarding the general role of autophagy in NAFLD development. More studies are necessary to clarify what molecular pathways influence molecules involved in autophagic degradation to switch from lipolysis to lipogenesis under varied nutritional status.

## 3. Molecular Mechanisms and Regulation of Impaired Autophagy in NAFLD

Decreased expression of autophagy-related genes, an impaired fusion of the autophagosome with the lysosome, and reduced levels of lysosomal enzyme production are the indicators of defective/impaired autophagic flux [45–49]. The role of ATG5 and other ATG proteins in the modulation of canonical, noncanonical, and defective autophagy has been recently reviewed [50]. These reports indicate that ATG5, a core component of autophagy, acts as a guardian of immune integrity, and dysregulation of ATG5 impaired the autophagic process in a variety of diseases including autoimmune diseases, autoinflammatory diseases, type 2 diabetes, Crohn's disease, and liver diseases [50]. The role and regulation of impaired autophagic function in fatty livers have been mostly studied in animal models and correlate with human NAFLD (Table 1). Zhao et al. observed the reduction of the expression autophagy markers LC3 and Beclin-1 after 1 h reperfusion in human liver allograft biopsies [51]. Downregulation of autophagy was positively correlated with increased hepatic steatosis and poor survival of liver transplant recipients. The study confirmed this observation using an animal model and demonstrated that the expression of ATG3 and ATG7 decreased in fatty liver due to higher expression of Calpain 2 protease during ischemia-reperfusion (I/R) [51]. Calpain 2-mediated degradation of ATG3 and ATG7 increased sensitivity to I/R-mediated liver injury [51]. Furthermore, downregulation of ATG7, ATG5, Beclin1, and LC3 and elevated levels of p62 and ER stress were shown to contribute to increased insulin resistance in *ob/ob* mice [40]. Mice fed an overnutrition diet displayed increased liver amino acid concentrations, induction of hepatic steatosis, and activation of mTOR, leading to impaired autophagic function in mouse liver [52]. Mice fed with a high-fat diet displayed increased insulin resistance and hyperinsulinemia, leading to downregulation of the expression of autophagy-related genes, thus indicating that hyperinsulinemia might contribute to impaired autophagy in diabetic *ob/ob* mice [53]. Inami et al. demonstrated that steatotic hepatocytes isolated from *ob/ob* mice showed impaired autophagosomal acidification and decreased expression of cathepsin B and L proteases in autolysosomes, leading to suppressed autophagic proteolysis [54]. An increase in p62 expression, accumulation of autophagosomes, and suppression of degradation of long-lived proteins (markers of impaired autophagy) without disturbing the fusion of autophagosomes with lysosomes was also observed in hepatocytes isolated from obese mice [54]. The study further pointed out that autosomal and liver cathepsin B and L proteinase activities were suppressed in *ob/ob* mice, indicating that hepatic steatosis inhibited autophagic proteolysis via impairment of autophagosomal acidification and cathepsin downregulation [54]. Moreover, in NAFLD patients, hepatic steatosis showed a decreased expression of cathepsin B, D, and L proteases leading to inhibition of autophagic proteolysis [55]. The increased number of autophagic vesicles was observed in NAFLD and chronic hepatitis C hepatocytes of liver biopsy specimens from patients with chronic liver diseases but not in chronic hepatitis B or primary biliary cirrhosis samples compared with control. Aggregation p62 was observed in 65% of NAFLD patients, which is correlated with serum alanine aminotransferase expression and inflammatory activity suggesting that hepatic inflammation is associated with autophagic impairment in NAFLD [55].

In NAFLD, several factors including temperature, ATP concentration, amino acid concentration, calcium concentration, alteration of autophagosome-lysosome lipid membrane composition, autophagosome acidification, and defective autophagosome-lysosome fusion can result in defective autophagy in the liver [56]. Koga et al. suggested that a variation in autophagosome-lysosome membrane lipid composition can deregulate autophagosome-lysosome fusion, particularly in animals fed with a high-fat diet or in obese animals [57]. Moreover, exposure of murine primary hepatocytes and murine normal BNL-CL2 cells or HCC-HepG2 cells with a saturated palmitic fatty acid increased expression of Rubicon (a Beclin-1-interacting negative regulator protein), increased expression of p62 and LC3-II, and decreased autophagosome-lysosome fusion, leading to inhibition of autophagy at later stages [58]. Similar results were also observed in mice fed with a high-fat diet or patients with NAFLD that showed increased expression of Rubicon, resulting in suppression of the autophagic process in liver tissues [58]. Furthermore, supplementation of palmitic fatty acid inhibited hepatic ATP2A2/SERCA2, an ER-calcium pump that is responsible for the influx of cytosolic calcium into the ER lumen [59]. Inhibition of this ER-calcium pump by palmitic acid increased cytosolic calcium levels effectively, affecting autophagic function in mouse liver [59].

Defective lysosomal acidification and impaired autophagy were also detected in NAFLD murine models [60]. This work suggested that ER stress-mediated expression of asparagine synthetase (ASNS) increased asparagine in the liver, affecting both calcium levels and lysosomal acidification [60]. Interestingly, studies also revealed that patients with severe NAFLD and HCC show decreased expression of glycine N-methyltransferase (GNMT) enzymes, leading to increased levels of serum methionine and its metabolite S-adenosylmethionine [61]. Also, treatment of hepatocytes with methionine and S-adenosylmethionine activates protein phosphatase 2A (PP2A) by methylation, leading to impaired autophagic catabolism and increased liver cell steatosis [61]. Further, dysregulation of lipid-metabolizing enzymes also plays an important role in NAFLD development by contributing to impaired autophagic function [62]. Genetic ablation of phospholipase D1 (PLD1) decreased the expression of lipid-metabolizing enzymes (Cpt1a, PPAR-*α*, Acat1, AcadV1), reduced lipid oxidation, and increased mitochondria swelling in the liver, resulting in increased hepatic triglyceride content and liver weight in mice fed with high-fat diet [62]. Mechanistically, *Pld1*^−/−^ hepatocytes show increased expression of LC3 I/II and p62 and accumulation of ubiquitylated proteins compared with *Pld1*^+/+^ hepatocytes. Treatment of *Pld1*^+/+^ hepatocytes with lysosomal protease inhibitors E64D and pepstatin A (PepA) show increased levels of LC3 I/II in the serum-fed or serum-starved cells. However, increased LC3-I/II expression was not observed in *Pld1*^−/−^ hepatocytes upon serum-fed/serum starvation or after E64D and PepA treatments, suggesting that *Pld1* deficiency regulates impaired autophagic flux and enhances hepatic steatosis [62].

**Figure 2:**
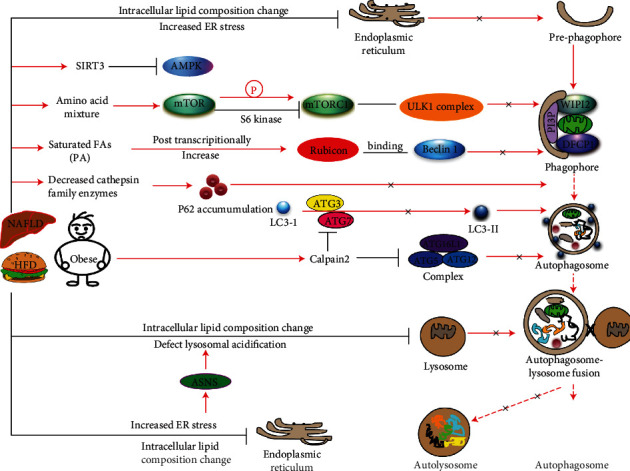
Schematic model represents the molecular mechanisms and dysregulation of autophagy components in a high-fat diet (HFD)/obesity-induced impaired autophagic function in NAFLD. HFD-/obesity-mediated activation of SIRT3 inactivates AMPK, mTORC1, and ULK1 complex leading to inhibition of phagophore formation. A high fatty acid diet upregulates Rubicon expression, increasing its interaction with Beclin 1 and decreasing autophagosome-lysosome fusion. HFD/obesity decreases the expression and activities of cathepsin family enzymes and downregulates autolysosomal proteolysis. HFD/obesity induces changes in the membrane lipid composition of the lysosome, affects autophagosomal-lysosomal acidification, and inhibits fusion of the autophagosome with the lysosome.

Impaired autophagy is regulated by several upstream and downstream kinases during NAFLD progression (Figure 2). Mice fed with a saturated fatty acid-rich high-fat diet showed increased expression of SIRT3 (nicotinamide adenine dinucleotide-dependent deacetylase) [63]. SIRT3 inactivates AMPK1 and overactivates mTOR, suppressing autophagy and increasing lipotoxicity in mouse liver [63]. Consistent with this observation, saturated fatty acids also induced lipotoxicity in NASH via activation of TANK-binding kinase 1 (TBK1) [64]. TBK1 phosphorylates p62, enhancing aggregation of ubiquitylated proteins and formation of large protein inclusions in hepatocytes, and induced lipotoxicity in mouse liver. Interestingly, inhibition of TBK1 in mouse liver by the TBK1 inhibitor BX795 reduces p62 inclusions, production of ROS, and liver fibrosis during NASH. In this context, TBK1 may control autophagic flux through p62 phosphorylation and induce lipotoxicity in the mouse liver [64].

A blockade of autophagic flux increased intracellular fat accumulation, both in patients with NAFLD and in a murine model of NAFLD, which was associated with elevated ER stress and defective autophagic flux [37]. Indeed, the same study demonstrated that in human NAFLD/NASH patients, there is an increased level of hepatic endoplasmic reticulum (ER) stress markers such as ATF4 (activating transcription factor 4), GRP78 (glucose-regulated protein 78), CHOP (C/EBP homologous protein), and impaired autophagic marker p62/SQSTM1 (p62)[37]. Liver samples from mice fed with a high-fat diet showed increased activation of ER stress signaling, as well as defective autophagic flux. Therefore, ER stress in parallel with impaired autophagic flux resulted in lipid-overload in hepatocytes, enhancing hepatic cell apoptosis [37]. Moreover, during ER stress the expression of *α* isoform of the inhibitor of Bruton's tyrosine kinase (IBTK*α*), a member of the unfolded protein response (UPR), specifically localized ER and by association with LC3B, SEC16A, and SEC31A proteins, induces phagophore initiation from ER exit sites. Exposure of saturated free fatty acids and IBTK*α* knockdown prevented accumulation of autophagosome intermediates stemming in hepatocytes. The study further suggested that during ER stress, induction of IBTK*α* and UPR inhibits autophagic flux that was associated with steatosis to NASH transition in NAFLD patients [65].

Transcription factor EB (TFEB) is known to regulate lipid metabolism and lysosomal biogenesis by upregulation of autophagic gene expression [66]. Although the role of TFEB activation and modulation of autophagic function is predominately observed in alcoholic fatty liver disease (AFLD), Wang et al. recently identified three drugs/compounds, specifically digoxin (DG), ikarugamycin (IKA), and alexidine dihydrochloride (AD), by quantitative high-throughput cell-based assay. These compounds activate and increase the nuclear localization of TFEB via three distinct Ca^2+^-dependent mechanisms [67]. The study further revealed that mice exposed to these small molecule agnostics of TFEB show a reduced high-fat diet-induced hepatic steatosis, which is associated with the upregulation Ppargc1*α*, Ppar1*α*, and Fgf21, a lipid metabolic enzyme expression. They also observed improved glucose and insulin tolerance and reduced p62/SQSTM1 accumulation in hepatocytes, suggesting that activation of TFEB by DG, AD, and IKA enhanced autophagic flux in mice fed with high-fat diet [67]. The role of TFEB in the modulation of impaired autophagic function in NAFLD remains to be elucidated.

Although there are no established biomarkers currently approved for clinical usage, impaired autophagy can be monitored by different markers such as increased expression of p62, accumulation autophagosomes, increased apoptosis, increased inflammation, a defective fusion of the autophagosome with the lysosome, and decreased autophagic proteolysis (Figure 2). Overall, these findings suggest that defective/impaired autophagy contributes to NAFLD progression, and several upstream and downstream kinases can affect autophagic flux in NAFLD.

## 4. Role and Regulation of Autophagy in AFLD

Acute and chronic alcohol consumption can result in liver injury since this organ is the primary site of alcohol metabolism [68]. Several enzymes, including alcohol dehydrogenase (ADH), aldehyde dehydrogenase (ALDH), cytochrome P4502E1 (CYP2E1), and catalase (peroxisomal), metabolize alcohol and generate acetaldehyde and acetate as a result [68]. Acute and chronic consumption of ethanol increases reactive oxygen species (ROS) production and oxidative stress and decreases antioxidant levels in many tissues, especially in the liver, leading to injury [69]. Excessive consumption of alcohol produces a wide spectrum of hepatic lesions that can eventually progress from hepatic steatosis (fat deposition) to hepatitis, fibrosis/cirrhosis, and HCC [70]. Heavy drinking or silent alcohol consumption induces nearly 80 to 90% of hepatic steatosis in the initial stages of AFLD. Out of these, 20 to 40% of patients with hepatic steatosis progress to steatohepatitis. During steatohepatitis, deposition of the extracellular matrix occurs in the liver resulting in liver fibrosis, and 8 to 20% cases of active fibrotic response progress to cirrhosis (liver scarring or liver failure). Unfortunately, if liver damage is not effectively addressed, 3-10% of these cirrhotic livers will progress to HCC [71].

**Figure 3:**
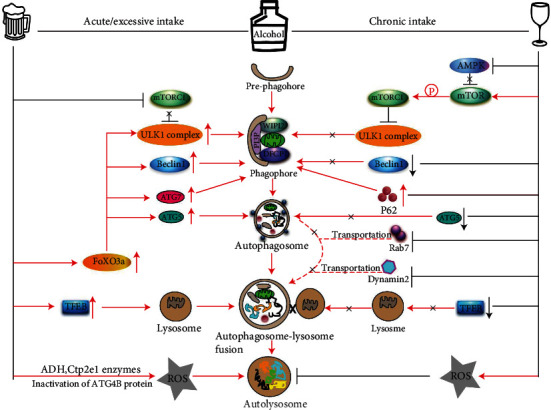
Schematic model represents the molecular role of acute and chronic intake of alcohol in the induction of autophagy/impaired autophagy in AFLD. Acute ethanol induces ADH- and CYP2E1-mediated ROS production that inactivates ATG4B protein and induces autophagy. Acute consumption of alcohol inhibits AKT and mTORC1 complex and increases FoxO3a- and TFEB-mediated expression of ATG5, ATG7, Beclin 1, and ULK1 proteins upregulating autophagosomal-lysosomal fusion and functional autophagy. In contrast, chronic intake/consumption of alcohol inactivates AMPK but activates the mTORC1 complex which in turn inactivates the ULK1 complex and inhibits phagophore formation. Moreover, chronic consumption of alcohol downregulates the nuclear localization of TFEB, reduces expression of Beclin-1, and ATG5 that inhibits the phagophore to autophagosome transition. Chronic alcohol also inhibits Dynamin 2, disturbs the transportation of Rab7 into the lysosomal membrane, and impairs autophagosomal-lysosomal fusion.

Excessive consumption of alcohol can also cause defective lipid export from liver tissues [72]. Reports suggest that ethanol can cause the accumulation of lipid droplets in the liver and can also dysregulate mitochondrial homeostasis, both events critically regulated by autophagy [48]. In AFLD, acute and chronic alcohol consumption increases oxidative stress and differentially regulates hepatic autophagic flux [73]. Hepatic autophagy is activated in acute ethanol consumption to aid in the metabolism of this molecule and avoid liver injury but can be suppressed during chronic or heavy alcohol consumption (Figure 3) [74, 75]. Acute ethanol consumption produces ROS, activates autophagy regulatory proteins, increases the number of autophagic vacuoles, and promotes autophagosome-lysosomal fusion (Figure 3). To support this, Ding et al. demonstrated that acute treatment of primary hepatocytes or hepatic cell lines with ethanol increased autophagosome formation and induced autophagy flux [76]. In our laboratory, recently, we demonstrated that acute exposure of ethanol to HCC cell lines activated autophagy as well as cell steatosis [77]. Similar observations were made in livers of mice fed a binge ethanol diet (4–6 g ethanol/kg body weight) [73, 76]. Acute ethanol-fed mice showed higher hepatic nuclear content of transcription factor EB (TFEB), which is considered to be a master regulator of transcription of genes involved in autophagy progression and lysosome biogenesis [73, 78]. Moreover, autophagy induced by adiponectin in liver cancer cells prevented ethanol-mediated cell apoptosis by regulation of AMPK/FoxO3 signaling and reduced acute ethanol-induced hepatotoxicity and steatosis in mice. These findings suggest that induction of autophagy may play a protective role against acute alcoholic liver damage [79].

Acute ethanol treatment triggered autophagy through the production of ROS, mediated by ADH and CYP2E1 enzymes, and by inactivation of the ATG4B protein [80]. Acute ethanol treatment also inactivated the mTOR complex, and inactivation of mTORC1 resulted in the activation of downstream ULK1 complex leading to the induction of autophagy [76]. Acute ethanol exposure inhibited AKT resulting in dephosphorylation and nuclear translocation of FoxO3a [81]. In the nucleus, FoxO3a bound to the promoter regions increased the expression of several autophagy-related genes, including *ATG5*, *ATG7*, *Beclin 1*, and *ULK1*, promoting autophagy in the mouse liver [81] (Figure 3). Moreover, treatment with resveratrol, a SIRT1 agonist, deacetylated FoxO3a and, further, increased autophagy-related gene expression in mouse liver induced by acute ethanol treatment [81]. Acetylation of FoxO family members attenuated their transcriptional activity and reduced autophagy-related gene expression [82, 83]. On the contrary, ethanol-induced autophagy increased the expression of PIAS family protein PIASy, which is involved in the accumulation of SUMO1-conjugated proteins, and enhanced HCV replication in hepatoma cell lines [84]. Indeed, these findings suggest that acute alcohol exposure increases autophagic flux in the liver and may prevent AFLD progression. However, a complete understanding of the molecular mechanisms underlying these observations remains unclear.

## 5. Mechanism and Regulation of Impaired Autophagy in AFLD

Impaired autophagic function predominantly exists in chronic alcohol intake. Chronic alcohol intake induced the production of ROS and suppression of hepatic autophagy thus promoting liver steatosis [85]. This process can be reversed by activation of mitochondrial aldehyde dehydrogenase 2 (ALDH2) which detoxifies acetaldehyde, a metabolic product of alcohol [85]. The effects of acute and chronic alcohol intake-mediated activation or suppression of autophagy were found to be dependent on the rate of alcohol metabolism, ROS production, and regulation of autophagy-controlling upstream factors such as mTORC1 and AMPK [48]. Studies show that during chronic AFLD, autophagy can be suppressed as demonstrated in mice fed with a Lieber-DeCarli diet for 4 weeks [86]. The study showed increased autophagy at a low dose of ethanol (accounting for 29% of the caloric intake), and significant inhibition of autophagic function in mice liver fed with a higher dose of ethanol (accounting for 36% of the caloric intake) [86]. Mechanistically, mice fed a chronic alcohol diet exhibited decreased nuclear translocation of transcriptional factor EB (TFEB) in the mouse liver [73], and decreased levels of TFEB expression in human ALD liver samples were also noted, indicating decreased autophagy [75]. Further, chronic ethanol exposure to hepatic cells or mice activates sterol regulatory element-binding protein 1 (SREBP-1) which modulates fatty acid synthesis and metabolism [87]. Chronic ethanol exposure inhibited AMP-activated protein kinase (AMPK) activity, increased acetyl-CoA carboxylase (ACC) activity, and enhanced malonyl CoA content, suggesting that inhibition of AMPK and downstream autophagic flux by ethanol promoted AFLD development in mouse liver [87]. On the other hand, exposure to chronic ethanol activates mTORC1 and decreased TFEB-mediated lysosomal biogenesis, resulting in suppression of autophagic processes in mouse livers [74].

Chronic ethanol consumption not only modulates AMPK/mTOR and TFEB but also affects the expression of autophagy core components. For example, liver tissues obtained from rats fed a chronic ethanol diet showed decreased expression of Beclin-1 and ATG5 and increased expression of p62 (a marker of defective autophagy) indicating liver injury [88]. The role of chronic ethanol exposure and suppression of lipophagy in rats has been also described [89]. These studies revealed that chronic ethanol treatment-induced hepatic steatosis, inactivated Rab7 (a small guanosine triphosphatase), and dynamin 2 activity, leading to impaired transport of Rab7 to the lysosomes [89, 90] (Figure 3). Additional studies demonstrated that rats fed with a chronic alcohol diet showed a significant decrease in the observed number of lysosomes and lysosomal functions in their livers [86]. Rat fed a liquid diet containing ethanol showed 20-50% decreased activities of lysosomal acid phosphatase and beta-galactosidase, as well as a reduction in intralysosomal hydrolase activity. Administration of an ethanol diet shifted these enzymes from the lysosomes to lower density cellular compartments. The study further pointed out that ethanol administration also increased the level of cathepsin L precursor (39 kDa) compared with its intermediate (30 kDa) or its mature product (25 kDa) suggesting that ethanol causes major impairment in the processing of procathepsin L to mature enzyme and thus reduced lysosomal proteolysis [91] (Table 1). During chronic AFLD, liver injury is worsened when autophagy is impaired, whereas the induction of normal and functional autophagy greatly improves liver condition [86]. The development of Mallory-Denk bodies (MDBs), which mainly consist of ubiquitin, p62, and keratin, is characteristic of chronic AFLD [92]. Studies demonstrate that during chronic AFLD, impaired autophagic function results in the accumulation of MDBs in the liver. Inhibition of mTOR (a negative regulator of autophagy) by rapamycin greatly reduced the number of MDBs, indicating that autophagy is necessary to clear MDBs from the liver [28, 92].

In summary, the evidence presented above indicates that chronic alcohol consumption impairs autophagy leading to alcoholic liver damage. On the other hand, acute consumption of alcohol activates autophagic flux preventing liver damage (Table 1 and Figure 3). Among other factors already driving excessive alcohol consumption in the U.S., recent studies show that there has been an increase in alcohol consumption during the COVID-19 pandemic [93]. It is imperative to understand how functional autophagy aids the liver to avoid long-term damage. Specifically, more in-depth studies are necessary to fully understand the effects of acute and chronic alcohol consumption in alterations to the liver architecture, induction of hepatic ROS levels, rate of alcohol metabolism, and the dysregulation of the autophagic flux, particularly in chronic AFLD. We also need additional experimental evidence related to how to restore autophagy therapeutically to prevent liver damage.

## 6. Regulation and Mechanism of Impaired Autophagy in HCC

The dual role of autophagy, both as a tumor suppressor and a promotor, has been studied in several cancers, highlighting the significance of this catabolic process in carcinogenesis [94, 95]. As shown in previous sections, impaired autophagic function exacerbates both NAFLD and AFLD and may contribute to the development of HCC. However, autophagy can also promote tumor cell survival in the presence of stressors [96]. Impaired autophagic functions can affect HCC progression, resulting in increased oxidative stress, mitochondria damage, and suppressed synthetic lethal deficiency in DNA repair, leading to chronic tissue damage and genome mutations in HPCs (hepatic progenitor cells) [96]. Dysregulation of autophagy-related proteins, such as p62, glypican-3, ATGs, LC3, and Rab7, was reported as a contributor to HCC development and progression [97, 98]. Furthermore, expression of p62 (100%) and glypican-3 (70%) were found to be upregulated in HCC tumor samples, whereas no p62 and glypican-3 were detected in matching controls, indicating impaired autophagic flux in these sets of HCCs samples [99]. Similarly, increased expression of p62 and glypican-3 has observed in hepatoma Huh-7.5 cells, indicating impaired autophagic flux, and activation of functional autophagy in Huh-7.5 cells efficiently cleared p62 and glypican-3 expression [99]. These results suggested that both p62 and glypican-3 expression may be utilized as markers of dysregulated autophagic function in HCC. Furthermore, Tian et al. recently demonstrated that impaired autophagy is involved in hepatocarcinogenesis. Hepatocyte-specific knockout of *ATG5* increased oxidative stress, and DNA damage leading to initiation of hepatocarcinogenesis [100]. The study further pointed out that wild-type mice exposed to diethylnitrosamine, a carcinogen, developed only benign tumors. However, *ATG5* knockout mice exposed to this compound developed multiple HCC tumors, suggesting that impaired autophagic function negatively regulates tumor suppressor p53 protein and that impaired autophagy is the main driver of the initiation of HCC tumorigenesis [100].

The dysregulation of essential autophagic genes, such as *ATG7*, *ATG5*, or *Beclin 1*, plays a key role in the occurrence and development of HCC, but the exact mechanisms are highly controversial. The expression of p62 is required for HCC induction and recurrence in mice, leading to the activation of Nrf2, mTOR, and cMyc and protection of HCC-initiating cells from oxidative stress-induced death [101]. Higher expression of p62 (an impaired autophagy marker) was observed in HCC tissues, and its expression positively correlated with the expression of fetal marker alpha-fetoprotein (AFP) and stem cell marker delta-like 1 homolog (DLK1) [102]. Transgenic expression of p62 in animal models shows an aggressive phenotype and increased susceptibility to chromosomal aberrations in HCC tumors [102]. Overexpression of p62 was observed in premalignant liver diseases and HCC [102]. Another report suggested that human antigen R increases mRNA expression of *ATG5*, *ATG12*, and *ATG16*, thus acting as a pivotal regulator of autophagosome formation [103]. Increased expression of human antigen R and ATGs can effectively lead to defective autophagy in HCC cells [103]. Moreover, impaired autophagy caused by the deficiency of *ATG5* and *ATG7* genes resulted in benign liver adenomas in mice [104]. Deletion of *ATG5* and *ATG7*, increased p62 expression, mitochondrial swelling, oxidative stress, and genomic damage in primary hepatocytes and deletion of the *p62* gene reduced tumor size in *ATG7* deficiency liver tumors [104]. Deletion of the autophagy regulatory gene *Beclin 1* in mice resulted in a higher incidence of spontaneous tumors, including HCC [105]. Beclin-1 facilitated the interaction of protooncogene avian myelocytomatosis virus oncogene cellular homolog (cMyc) and its phosphatase PP2A, leading to dephosphorylation and degradation of c-Myc, resulting in decreased cell division and cancer cell proliferation [106]. Deregulation of c-Myc correlated with increased tumorigenesis in Beclin-1 defective systems, suggesting that Beclin-1 acts as a haploinsufficiency tumor suppressor gene in cancer [106] (Table 1). Furthermore, autophagy regulators BECN1 and PIK3C3 interact with UVRAG (UV radiation resistance-associated), and UVRAG ubiquitination at its K517 and K559 residues and promoted autophagosome maturation as well as lysosomal degradation of epidermal growth factor receptor (EGFR), leading to the inhibition of HCC cell growth [107].

Autophagy and chaperone-mediated autophagy (CMA) are two distinct and major lysosomal degradation processes that compensate for each other to facilitate HCC cell survival [108]. Cirrhotic livers with HCC showed increased expression of p62 (84%) and glypican-3 (78%), while adjacent nontumorous hepatocytes showed decreased expression of these proteins, suggesting that impaired autophagic flux exists in cirrhotic livers with HCC. This study further pointed out that altered expression of LAMP-2A was observed in 95% of HCCs which is associated with induction of CMA [108]. Also, inhibition of lysosomal degradation increased p53 and cell apoptosis, whereas activation of autophagy by mTOR inhibition promoted HCC growth, suggesting that CMA compensates for the impaired autophagy to promote HCC survival in the cirrhotic liver [108].

Autophagy has also been demonstrated to enhance cancer cell survival after exposure to antineoplastic agents or other stressors. Treatment of hepatoma cells with the antineoplastic tyrosine kinase inhibitor drug sorafenib inactivated mTORC1 resulting in increased accumulation of autophagosomes, LC3B II, and autophagic flux, whereas *ATG7* knockdown sensitized hepatoma cells to sorafenib [109]. Moreover, sorafenib increased autophagic flux in Huh7 xenograft tumors in nude mice as demonstrated by increased LC3B II activation. Interestingly, inhibition of autophagic flux by chloroquine and subsequent treatment with sorafenib suppressed tumor growth compared with sorafenib exposure alone, suggesting that sorafenib treatment increased autophagic flux in hepatocytes, giving these cells a survival advantage that can result in tumor reoccurrence. On the other hand, inactivation of autophagy together with sorafenib treatment suppressed HCC tumor growth, highlighting the significant role of this catabolic process in this malignancy [109].

The Hippo pathway, a highly conserved pathway that regulates cell proliferation, stem cell renewal, and cell survival, plays an important role in HCC suppression by restricting Yap activation, tissue overgrowth, and carcinogenesis [110]. A recent study suggests that the Hippo pathway effector protein Yap degrades through autophagy [111]. Lee et al. demonstrated that hepatocyte-specific deletion of *ATG7* promotes liver size, fibrosis, progenitor cell expansion, and hepatocarcinogenesis through Yap stabilization. Knockdown of Yap in *ATG7* knockout mice resulted in decreased hepatomegaly, altered liver tissue architecture, and decreased hepatocarcinogenesis compared with ATG7 knockout mice, suggesting that both Yap degradation and impaired autophagic function can disrupt HCC progression [111]. Furthermore, higher expression of LC3 is significantly associated with vascular invasion and lymph node metastasis in HCC patients, suggesting that autophagy may promote malignant progression [112]. Also, a meta-analysis of HCC cases revealed that increased LC3 expression correlated with tumor number, tumor size, liver cirrhosis, TNM stage, vascular invasion, histological grade, expression of alpha-fetoprotein, and HBsAg [113]. The dual role of autophagy, as both a tumor suppressor and a tumor promotor, is summarized in Table 1. Overall, these studies suggest that impaired autophagy is involved in HCC progression.

## 7. Selective Autophagy and Liver Diseases

Although autophagy was previously thought to be a nonregulated process of cell catabolism, it has now been shown to be a highly regulated process with physiologically diverse functions. The role of selective autophagy in the modulation of liver physiology, liver injury, fatty liver diseases including NAFLD, AFLD, viral hepatitis, and liver cancer has been recently reviewed [22]. Aggrephagy, lipophagy, mitophagy, and xenophagy can occur concurrently in liver disease, and their activation is mainly dependent on specific pathological conditions, including stress, starvation, and liver injury [17, 114–117]. Earlier studies suggested that selective aggrephagy controls the turnover of intracellular macromolecules and that amino acid and insulin deprivation resulted in autophagic degradation of protein and RNA molecules in perfused rat liver samples [118]. Degradation of proteasomes by lysosomes was also observed in rat livers when animals were starved for 24 h, suggesting that functional autophagy can control macromolecule turnover by downregulation of the ubiquitin–proteasomal pathway [115]. Degradation of Mallory–Denk bodies (MDBs) [119, 120], lipid droplets [26, 29], peroxisomes [121–123], mitochondria [124, 125], and ER [40, 126] by selective autophagy have all been reported. Impaired lipophagy increased the NAFLD activity score (NAS) along with fibrosis in both mice fed with a high-fat diet and in NAFLD patients [38]. Mitophagy predominantly exists in ALD, and consumption of alcohol can inhibit the synthesis of mitochondrial respiratory complex proteins resulting in mitochondrial dysfunction [127]. Also, ethanol reduced oxidative phosphorylation, increased ROS production, mitochondrial DNA damage [128], and impaired mitophagy [129, 130]. This resulted in an inability to degrade damaged mitochondria, promoting ALD pathogenesis [129, 130]. Animal model studies indicate that compounds like quercetin and wolfberry-derived zeaxanthin dipalmitate can restore mitophagy in ALD and prevent liver injury [131, 132]. Moreover, hepatic xenophagy is known to modulate HBV and HCV infection, and an earlier study also demonstrated that HCV-H77 (genotype 1a) infection induced autophagic vacuoles in immortalized human hepatocytes [133]. HCV viral RNA replication impaired autophagosome formation in human HCC and Huh7 cells, thus dysregulating autophagic flux [134]. Moreover, studies demonstrated that nonstructural (NS) proteins of HCV, NS4B, and NS5B interact with autophagy core component ATG5 to promote HCV replication [135]. Furthermore, autophagosomal membranes provide a platform for HCV replication [136, 137], suggesting that viral HCV infection activates autophagy in viral replication. Interestingly, other reports indicate that HCV-induced autophagic vacuole formation does not allow the colonization of viral proteins or RNA, suggesting that viral infection-mediated induction of autophagic vacuole formation may not provide a platform for viral RNA replication [138, 139]. Indeed, selective autophagy plays an important role in the regulation of metabolic pathways, as well as in the elimination of damaged organelles, protecting liver cells from injury. Impairment of selective autophagy results in increased liver toxicity and NAFLD, AFLD, and HCC progression.

## 8. Autophagy Inhibitor/Activator and Liver Diseases

Since both functional autophagy and impaired autophagy play roles in NAFLD, AFLD, and HCC development and progression, targeting this catabolic process could represent a new therapeutic strategy to improve disease prognosis. Several inhibitors of autophagy, such as SBI-0206965, MRT68921, and MRT67307 (which mainly target ULK1, an enzyme involved in membrane nucleation, an initial stage of autophagy), have successfully been utilized *in vitro* to decrease autophagic function. However, there are no current reports of their utilization or effectiveness in vivo models [140, 141]. Common autophagy inhibitors, like 3-methyl adenine (3-MA), LY294002 [2-(4-morpholinyl)-8-phenyl-4H-1-benzopyran-4-one hydrochloride], and wortmannin, target Class III PI3K and Vps34 complexes resulting in decreased formation of autophagosomes [142]. Inhibition of lysosomal acidification also suppressed autolysosome formation, and protease inhibitor E64d inactivated Pepstatin A and Cathepsin activity, inhibiting autophagic function in later stages [143]. Similarly, chloroquine and hydroxychloroquine effectively inhibit lysosomal acidification by increasing pH inside the lysosome [142, 144].

The development of therapeutic regimes in combination with inhibitors or inducers of autophagy is being explored for diseases such as cancer, as recently reviewed by Liu et al. [145]. Drugs such as chloroquine and hydroxychloroquine were recently utilized in Phase I/II clinical trials as autophagy inhibitors in patients with various types of cancers. Administration of chloroquine and inhibition of ATG5 by short hairpin RNA activated p53 and increased p53-mediated cell apoptosis in a Myc-induced model of lymphoma generated from cells derived from p53ER(TAM)/p53ER(TAM) mice [146]. The role of autophagy inhibitors chloroquine and hydroxychloroquine in preclinical and clinical development has been recently summarized [147]. Drugs such as the tyrosine kinase inhibitor linifanib or cytotoxic drugs such as doxorubicin in combination with autophagy inhibitors such as chloroquine, hydroxychloroquine, or 3-MA have also been explored for the treatment of HCC [145]. Hence, the utilization of autophagy inhibitors in combination with chemotherapeutic drugs may be an effective therapeutic strategy to improve patient prognosis at this stage since these agents might limit the ability of cancer cells to thrive under these extreme conditions [148]. However, additional studies utilizing these autophagy inhibitors in combination with current antineoplastic regimes for the treatment of advanced stages of cancer in a diverse group of patients are still needed to determine their safety and effectiveness.

Autophagy defects or impaired autophagy has been linked to a wide range of medical illnesses that include inflammatory diseases, infectious diseases, neurodegenerative diseases, cancer, and metabolic diseases like NAFLD and AFLD. In recent years, a novel concept has been developed which proposes that the utilization of compounds that induce autophagy might play an important role in the prevention or treatment of certain disease conditions [149]. Holistic approaches, such as a healthy lifestyle, a nutritious diet, and exercise, could promote health benefits through modulation of the autophagic pathway. Also, several FDA-approved drugs have been shown to enhance autophagic function. Therefore, autophagy-inducing drugs and compounds may have utility in clinical implications [149].

Impaired autophagy predominately exists in severe NAFLD, chronic AFLD, and HCC. The reconstitution of normal autophagic functionality could complement current and novel therapeutic regimens for the treatment of these liver diseases [150]. Improving autophagic function could be beneficial for both NAFLD and ALD patients, and earlier studies demonstrated that autophagy-enhancing pharmacological drugs and nutritional supplements reduced the risk of liver injury in NAFLD and ALD in murine models [48]. One of the first preclinical studies exploring the effectiveness of autophagy-inducing drugs showed that the autophagy inducers carbamazepine and rapamycin improve NAFLD and ALD pathology in mice [86]. Mice fed with HFD or chronic ethanol and injected with the combination of carbamazepine and rapamycin reduced hepatic steatosis and insulin resistance in mice. However, chloroquine alone increased steatosis and liver injury in these animals, demonstrating that pharmacological manipulation of autophagy was effective in reducing liver injury [86]. A low dose of wortmannin (a PI3K-AKT pathway inhibitor) decreased triglyceride levels in mice liver treated with acute ethanol. However, triglyceride levels increased in livers of mice given a high dose of ethanol, [151] suggesting that wortmannin differentially modulates autophagic function depending on levels of alcohol exposure in AFLD [151]. Several other mTOR inhibitors “rapalogs” such as Rottlerin, Torin, Z1001, PP242, XL388, AZD3147, AP23573, and RAD001 are known to induce autophagy, but these drugs have not been tested for clinical use [152]. Nutritional supplements including vitamin D, retinoic acid, caffeine, resveratrol, and glycycoumarin induced hepatic autophagy and reduced steatosis in mouse liver [153–155]. Vitamin D reduced HFD-mediated haptic steatosis by increasing expression of ATG16L1, an autophagy core component, whereas glycycoumarin inhibited lipoapoptosis by reactivation of impaired autophagy [155, 156], suggesting that autophagy inducer drugs/compounds can improve FLD pathology. Finally, induction of autophagy to potentially treat diseases using several pharmacological agents targeting autophagic function are currently being explored in Phase I/II clinical trials. For example, rilmenidine induces mTOR-independent autophagy showing improvement in Huntington disease clinical models [157, 158]. Administration of rilmenidine, an investigational small molecule, reduced levels of the mutant Huntingtin fragment in a mouse disease model [157] and also reduced cAMP levels in cellular models of the disease [158]. The role of rilmenidine in the reduction of hepatic steatosis was previously reported [159]. Rilmenidine modulates intracellular calcium ions by activation of the imidazoline I-1 receptor, increasing phosphorylation of p38 and the expression of nuclear receptor farnesoid X receptor (FXR), leading to reduced levels of hepatic steatosis in mice and cell steatosis in HepG2 HCC cells [159]. The testing of rilmenidine in FLD and HCC clinical trials has not yet been carried out.

Restoration of proper autophagic function, particularly in NAFLD, chronic AFLD, and HCC, may be an effective strategy to halt the progression of these liver diseases. Efforts to test these ideas, such as clinical trial NCT01988441 (as identified in http://clinicaltrial.gov; are currently exploring the role of autophagy and pathway-related genetic polymorphisms in developing the advanced liver disease (AdLD). Another trial, NCT03037437, is exploring the efficiency of the combination of sorafenib/hydroxychloroquine in the treatment of advanced HCC. However, to our knowledge, clinical trials specifically exploring the utilization of pharmacological agents that induce or inhibit autophagy have not yet been tested in the context of NAFLD or AFLD. Additional studies are necessary to develop targeted therapies to prevent and restore defective autophagy and demonstrate the effectiveness of the approach in a clinical setting.

## 9. Conclusions and Future Perspectives

**Figure 4:**
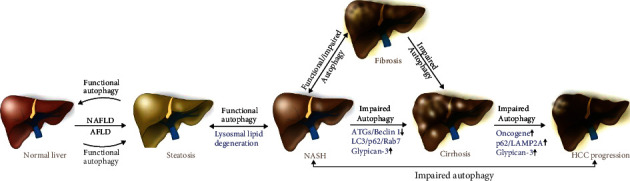
Schematic model represents the role of functional and impaired autophagy in the modulation of NAFLD, AFLD, and HCC. Functional autophagy can reverse hepatic steatosis, NASH, or hepatitis by the degradation of lipid droplets in the initial stages of NAFLD and acute AFLD. When a functional autophagic process is unable to process the overloaded-lipid content in the liver, particularly in chronic NAFLD and AFLD, defective autophagy exists. Due to defective/impaired autophagic function (increasing expression of p62, LC3, Rab7, and Glypican-3), NASH progresses to fibrosis/cirrhosis and subsequently to HCC.

In the current review, we summarized the role of impaired autophagy in liver diseases. An impaired autophagic process is strongly associated with the development of various human diseases such as Parkinson's disease, Crohn's disease, autoimmune diseases, diabetes, and cancers [160]. Here, we mainly focused on how impaired/defective autophagic function affects NAFLD, AFLD, and HCC development and progression (Figure 4). Defective autophagy is unable to degrade lipid droplets in the liver, resulting in the induction of hepatic steatosis, an early event in NAFLD or AFLD. Impaired autophagy affects NAFLD and AFLD and HCC progression and overall increases the severity of these diseases (Figure 4). Underlying causes of a defective autophagic process include (1) dysregulation of upstream-cell signaling cascades (AMPK, Class III PI3CK3, mTOR, and ULK1 complex), (2) dysregulation of autophagy core proteins (ATGs and Beclin), (3) inhibition of lipidation of LC3 (due to inhibition or oxidation of ATGs), (4) alteration of membrane composition of the autophagosome and lysosome (leading to impaired fusion of the autophagosome and lysosome), and (5) inhibition of proteolysis in the autolysosome (due to alteration of acidification in autolysosome). In NAFLD, defective autophagosome and lysosome fusion were observed to be mostly due to excessive lipid accumulation in hepatocytes. On the other hand, in chronic AFLD, excessive ROS production suppressed the development of the autophagosome as well as the fusion of autophagosome and lysosome. Since there is no definitive pharmacological treatment of NASH, the development of effective treatments by targeting autophagy would be a significant development. An increase in liver autophagy could be used as a potential therapeutic target in the treatment of NASH. Decreased cellular oxidative stress, better turnover of damaged ER and mitochondria, decreased triglyceride and cholesterol levels in hepatocytes, improved insulin homeostasis, would gradually decrease the chances of NAFLD, AFLD, and liver malignancy development [161].

Studies of the role of autophagy in these conditions are limited by the fact that there are no precise methods to determine the exact levels of autophagy in NAFLD patients [162]. Quantification of numbers of autophagosomes, or analyzing the expression of autophagy markers (ATGs, LC3, and Beclin1), is not a reliable factor to monitor autophagic function, because proteasomal degradation is also increased when the expression of these autophagy-related proteins is higher.

To tackle conditions involving impaired autophagy and related risks in liver diseases, identification of dysregulated autophagy-related genes/protein markers (SQSTM1/p62, Rab7 and LAMP proteins, autophagosome accumulation, and defective lysosomal function) is important to effectively restore functional autophagic processes (Figure 4).Identification of appropriate methods, identification of impaired autophagic biomarkers to assess autophagic flux in early-stage, and identification of autophagy biomarkers in the specific spectrum of liver diseases *in vivo* are also important steps for the development of autophagy-targeting strategies. Since NAFLD, AFLD, and HCC progress in stages and autophagy/impaired autophagy levels vary during disease progression, a proper method is needed to monitor and quantify the level of the impaired autophagy flux during the disease progression. Generally, in the early stages of liver disease, autophagy might be beneficial to control lipid accumulation. The occurrence of defective autophagy may increase as liver disease progresses and contribute to an increased pathological condition. Facilitating functional autophagy and inactivation of impaired autophagy by specific inhibitors is crucial to control hepatic steatosis to HCC transitions [163, 164]. Vector-based expression of autophagy regulatory proteins or treatments with specific doses of autophagy-targeted drugs could also be used to manage and normalize the autophagic processes in the initial stages of liver diseases. Importantly, since autophagy can also enhance cell survival in conditions of extreme cellular stress, such as chemotherapy, an appropriate stratification of patient “autophagy status” is important to effectively target this pathway. In this context, impairing autophagy with drugs such as hydroxychloroquine may be beneficial to the patient and could represent a novel therapeutic strategy.

In conclusion, the study of autophagy represents a relatively unexplored frontier in the development of disease biomarkers and alternative therapeutic regimes. However, due to its dual role in disease, specific markers that can effectively discriminate whether functional or impaired autophagy is the driver for disease progression still need to be developed for clinical utilization.

## Figures and Tables

**Table 1:** Impaired autophagic function in NAFLD, AFLD, and HCC.

Liver disease	Impaired autophagy function	Ref.
NAFLD	Hepatic steatosis in mice blocks autophagic proteolysis via impairment of autophagosomal acidification and cathepsin expression.	[54]

NAFLD	Animal fed with high-fat diet reduces autophagosome/lysosome fusion by 70% compared with animals fed with a normal diet.	[57]

NAFLD	In diabetic/ob/ob mice, HFD increased insulin resistance and hyperinsulinemia leading to impaired autophagic function caused by downregulation of autophagy regulatory gene expression.	[53]

NAFLD	Mice fed with HFD or patients with NAFLD show increased expression of Rubicon impairing the autophagic process in liver tissues.	[58]

NAFLD	Mice or hepatocytes treated with palmitic fatty acid show inhibition of ATP2A2/SERCA2, an ER-calcium pump, leading to increasing in cytosolic calcium levels and impaired autophagic flux due to interference calcium in the fusion of autophagosomes with lysosomes.	[59]

NAFLD	Hepatocytes treated with methionine and S-adenosylmethionine activated PP2A by methylation, leading to impaired autophagic catabolism and hence increased liver steatosis.	[61]

NAFLD	Genetic ablation of PLD1 in mice decreased the expression of Cpt1a, PPAR-*α*, Acat1, AcadV1, and increased impaired autophagic flux (p62 and LC3-II) and enhancing hepatic triglyceride accumulation in liver samples.	[62]

NAFLD	Mice fed with HFD containing saturated fatty acids, show increased SIRT3, inactivation of AMPK1, and activation of mTOR, leading to impaired autophagic function and increased lipotoxicity in the liver.	[63]

NAFLD/NASH	Hepatocytes exposed to saturated fatty acids showed increased activation of TBK1. TBK1 phosphorylated p62, inducing impaired autophagy leading to aggregation of ubiquitinated proteins and protein inclusions, increasing lipotoxicity in hepatocytes.	[64]

NAFLD	Mice fed with HFD or Huh7 hepatic cells treated with palmitic acid, showed increased expression of p62, LC3-II, and accumulation of autophagosomes, suggesting a defective autophagic flux.	[37]

NAFLD steatosis/NASH	ER stress enhanced IBTK*α*, LC3B, SEC16A, and SEC31A localization on ER and initiated phagophore formation, whereas, induction of IBTK*α* and inhibition of autophagic flux was associated with steatosis to NASH transition in NAFLD.	[65]

NAFLD	Increased autophagic vesicles and decreased cathepsin B, D, and L protease activities were detected in human NAFLD patients suggesting that hepatic steatosis induced impaired autophagy in the liver and reduced autophagic proteolysis.	[55]

AFLD	Chronic ethanol exposure activated mTORC1, downregulated TFEB-mediated lysosomal gene expression, and lysosomal biogenesis leading to defective autophagy in mice liver.	[74]

AFLD	Hepatic cells or mice chronically exposed to ethanol, show inhibition of AMPK, activation of ACC activity, and increased malonyl CoA content in liver tissues due to the suppression of autophagy.	[87]

AFLD	Rats fed with chronic ethanol downregulates Beclin-1 and ATG5 expression and upregulated p62 in liver tissues, indicating impaired autophagic function.	[88]

AFLD	Chronic ethanol exposure in rats inactivated Rab 7 and dynamin 2 activities resulting in impaired lipophagy.	[89, 90]

AFLD	Chronic ethanol exposure in rats decreased activities of lysosomal acid phosphatase, beta-galactosidase, and intralysosomal hydrolase activity in rat hepatocytes.	[91]

HCC	HCC tumors or HCC cells show higher expression of p62 and glypican-3 indicating defective autophagy liver cancer.	[99]

HCC	Impairing autophagy by *ATG5* knockdown in hepatocytes increased oxidative stress and DNA damage leading to initiation of hepatocarcinogenesis.	[100]

HCC	Human antigen R increased *ATG5*, *ATG12*, and *ATG16* transcripts leading to defective autophagy in HCC cells.	[103]

HCC	Deficiency of *ATG5* and *ATG7* genes induced impaired autophagy and increased development of benign liver adenomas in mice.	[104]

HCC	The deletion of the *Beclin 1* gene in mice impaired the autophagic process increasing the incidence of spontaneous tumors.	[105]

HCC	Treatment of sorafenib induced autophagy, whereas the deletion of *ATG7* impaired autophagic function leading to increased sensitivity of HCC cells towards antiliver cancer drugs.	[109]

## Abbreviations

FLD: Fatty liver disease
NAFLD: Nonalcoholic fatty liver disease
AFLD: Alcoholic fatty liver disease
HCC: Hepatocellular carcinoma
ROS: Reactive oxygen species
AKT: Protein kinase B
mTOR: The mammalian target of rapamycin
ER: Endoplasm reticulum
AMPK: AMP-activated protein kinase
ATGs: Autophagy-related genes
ULK1: Unc-51 like autophagy activating kinase: FIP200: FAK family kinase-interacting protein of 200 kDa
PI3K: Phosphatidylinositol 3-kinase
AMBRA1: Autophagy and Beclin1 regulator 1
VPS34: Vacuolar protein sorting 34
VPS15: Vacuolar protein sorting 15
UVRAG: UV radiation resistance-associated gene
LC3: Microtubule-associated protein 1A/1B-light chain 3
PE: Phosphatidylethanolamine
LAMPs: Lysosomal-associated membrane proteins
Rab7: Ras-related protein Rab-7
SIRT1: Sirtuin 1
FoxO3: Forkhead box protein O3
HCV: Hepatitis C virus
CoA: Coenzyme A
PP2A: Protein phosphatase 2
ERK/MAPK: Mitogen-activated protein kinase
PERK: Protein kinase R-like endoplasmic reticulum kinase
p62/SQSTM-1: Sequestosome 1
PI3P: Phosphatidylinositol 3-phosphate
SMURF1: Smad ubiquitin regulatory factor 1
cMyc: Avian myelocytomatosis virus oncogene cellular homolog
EGFR: Epidermal growth factor receptor
ASNS: Asparagine synthetase (glutamine-hydrolyzing)
ApoB: Apolipoprotein B
NASH: Nonalcoholic steatohepatitis
HFD: High-fat diet
S6K1: mTOR/S6 kinase
IRS1: Insulin receptor substrate-1
GNMT: Glycine N-methyltransferase
PLD1: Phospholipase D1
SFAs: Saturated fatty acids
TFEB: Transcription factor EB
CYP2E1: Cytochrome p450 2E1
ADH: Alcohol dehydrogenase
ALDH: Aldehyde dehydrogenase
EMT: Epithelial-mesenchymal transition
CaMKK-*β*: Ca^2+^/calmodulin-dependent kinase kinase-*β*.
